# Clinicopathological Features and Management of Cancers in Lynch Syndrome

**DOI:** 10.1155/2012/350309

**Published:** 2012-04-30

**Authors:** Markku Aarnio

**Affiliations:** Department of Gastroenterological Surgery, Jyväskylä Central Hospital, 40620 Jyväskylä, Finland

## Abstract

Lynch syndrome (LS) is characterized by an autosomal dominant inheritance of the early onset of colorectal cancer (CRC) and endometrial cancer, as well as increased risk for several other cancers including gastric, urinary tract, ovarian, small bowel, biliary tract, and brain tumors. The syndrome is due to a mutation in one of the four DNA mismatch repair (MMR) genes *MLH1*, *MSH2*, *MSH6*, or *PMS2*. The majority of LS patients and families can now be identified, and the underlying mutation detected using genetic diagnostics. Regular surveillance for CRC and endometrial cancer has proved beneficial for mutation carriers. However, screening for other tumors is also recommended even though experiences in the screening of these tumors is limited. Prophylactic colectomy, prophylactic hysterectomy, and bilateral salpingo-oophorectomy may be reasonable options for selected patients with LS. This paper describes the features and management of LS.

## 1. Introduction

Lynch syndrome (LS), also referred to as hereditary non-polyposis colorectal cancer (HNPCC), is the most common form of hereditary colorectal cancer, accounting for 2–5% of all colorectal cancer (CRC) cases [1, 2]. The cancer predisposition in LS arises from germline mutations in any of the four DNA mismatch repair (MMR) genes *MLH1*, *MSH2*, *MSH6*, or* PMS2* [3]. The mutation carriers are at high risk for developing CRC and endometrial cancer at a young age [4]. Many other tumor types such as gastric, ovarian, small bowel, urinary, and biliary tract, as well as brain tumors, have also been associated with LS [5, 6].

Clinical criteria, known as Amsterdam criteria I and II, have served to identify in LS families [7, 8]. Knowledge of the molecular genetic background of this syndrome has made it possible to develop molecular diagnostic methods, such as microsatellite instability (MSI) and immunohistochemical analysis, to identify cases with LS [9, 10]. Identification of these high-risk individuals is necessary so that screening programs can be initiated to prevent the development of cancers. Screening for colorectal and endometrial cancers has proved beneficial for mutation carriers of LS [11, 12]. Obtaining more data on the features and behavior of different tumors associated with LS is also important in order to develop the management of these tumors.

This paper reviews the clinicopathological features, diagnostic criteria, and management of LS.

## 2. Genetic Characteristics of Lynch Syndrome

Mutations in four DNA mismatch repair (MMR) genes, *MLH1*, *MSH2*, *PMS2*, and *MSH6*, are known to cause susceptibility to LS [3]. The main function of MMR is to maintain genomic stability by correcting mismatches generated during DNA replication. MMR malfunction results in a mutator phenotype and microsatellite instability (MSI). In mutation carriers with Lynch syndrome, one mutation is present in each copy of the gene inherited via the germline and is therefore present in all cells. Only one other mutation (somatic) is required for the development of cancer. Approximately 95% of mutations reported so far in LS families have been the *MLH1*, *MSH2*, or *MSH6* genes [13].

MSI is a hallmark of tumors in LS, but is also present in 15–25% of corresponding sporadic tumors [14]. Research has shown that the frequency of MSI is 60–100% in colorectal, endometrial, stomach, and uroepithelial cancers associated with LS [15–19]. MSI testing has therefore served as the diagnostic screening method for LS.

## 3. Identification of Lynch Syndrome

A family history of cancer plays an important role in the recognition of LS. Clinical criteria (Amsterdam I and II) have served in identifying LS families for clinical or research purposes [7, 8]. Amsterdam I criteria include the following: (1) at least three relatives should have histologically verified colorectal cancer, one of whom should be a first-degree relative to the other two; (2) at least two successive generations should be affected; (3) colorectal cancer should be diagnosed in one of the relatives under 50 years of age; (4) familial adenomatous polyposis should be excluded [6]. In 1999, the International Collaborative group on HNPCC introduced new selection criteria (Amsterdam criteria II) that also included extracolonic cancers associated with LS [8].

Clinical criteria known as the Bethesda Guidelines have been developed to enable identification of colorectal tumors in relation to which molecular analysis should be undertaken [9, 10]. A patient who meets the Bethesda criteria may need for molecular genetic studies either by MSI analysis of the tumor or immunohistochemical analysis of the MMR proteins. If a mutation is identified in the family, genetic counselling and genetic testing should be organized for all at-risk relatives. Such testing allows clinical screening to focus on mutation carriers and to exclude noncarriers from repeated examinations.

## 4. Tumor Spectrum of Lynch Syndrome

CRC and endometrial cancer are the most prominent types of cancer seen in LS [4]. However, several other cancer types have been shown to occur more frequently in LS than in the general population: cancers of the stomach as well as urological (uroepithelial and kidney), ovary, biliary tract, small bowel, and brain tumors [5, 6]. Researchers have suggested that cancer phenotypes associated with *MLH1*, *MSH2*, and *MSH6* gene mutations differ. For example, Vasen et al. [20] found a higher risk for extracolonic cancers in mutation carriers of *MSH2* than in those carriers of *MLH1*.

## 5. Colorectal Cancer

Colonic tumors are the most common tumors in LS, accounting for approximately 60–70% of the total [21, 22]. Studies have reported that a lifetime risk for developing CRC in mutation carriers is 70% at age 70 (range 30–80%) [5, 20, 23]. However, the estimation of risk seems to differ depending on sex and type of mutation. A Finnish study [5] found that the standardized incidence ratio for CRC was higher in men (83) than in women (48), and the male-to-female ratio was 1.7. The reason for the relatively low incidence of CRC in women is unknown. Dunlop et al. [23] suggested that women may be protected in some way because of environmental factors or even a sex-linked modifier gene. There is no difference in CRC risk between *MLH1* and *MSH2* mutation carriers. However, Hendriks et al. [24] observed that female *MSH6* mutation carriers are at significantly lower risk for CRC than are *MLH1* and *MSH2* mutation carriers.

Several clinical features typical of colonic tumors are associated with this syndrome. They most often occur in the proximal colon (60–70% in LS, 30% in sporadic CRC). CRC occurs at younger ages in HNPCC (mean 40–45 years) than in sporadic cases (mean 60–65 years), and mutation carriers of HNPCC are at higher risk for multiple synchronous and metachronous CRCs [21, 22].

Colorectal tumors in LS seem to evolve through the adenoma-carcinoma sequence, as they do in sporadic CRC [25]. In Ls, however, the adenomas occur in younger individuals and tend to be larger and more severely dysplastic than in sporadic cases [26–29]. In addition, studies have shown that adenomas in LS patients are located mainly in the proximal colon [29, 30]. Most adenomas in mutation carriers show MSI or the absence of immunohistochemical staining of the MMR proteins [31]. For CRC in LS, studies have reported MSI frequencies of 85–90% [16]. It has been suggested the that the adenoma-carcinoma sequence is accelerated in LS and that the progression from adenoma to carcinoma may take two to three years compared to eight to ten years in the general population [32]. Also, the histopathology of CRC in LS patients has some special features in comparison to CRC in general, as CRC is often mucinous and poorly differentiated [26, 27]. Diploid tumors and singlet-ring cancers also seem to be common in Lynch syndrome [26, 33]; however, it should be noted that, although these features are not specific to this syndrome, they could serve as additional markers for its recognition.

Colonoscopic surveillance has been recommended for mutation carriers of LS to prevent the development of cancer. Järvinen et al. [11] conducted a long-term controlled study comparing colonoscopic screening (three-year intervals) with no screening of at-risk members of LS families. The results showed that screening significantly reduced the incidence of CRC (6% in screened patients versus 16% in controls) and in overall mortality (8% in screened patients versus 22% in controls). Recently, a group of European experts in hereditary gastrointestinal cancer recommended screening by colonoscopy for mutation carriers every one to two years beginning at age 20 to 25 [34].

As mentioned above, mutation carriers of LS are at relatively high risk for synchronous or metachronous tumors. Therefore, researchers have suggested that total or subtotal colectomy is the operation of choice in LS patients with colorectal tumors [35]. Others have discussed prophylactic colectomy as a reasonable option in mutation carriers for whom colonoscopy is painful or difficult [36, 37]. In addition, prophylactic surgery may be the best treatment for a patient with several adenomas that cannot be removed endoscopically [36, 37]. Presently, however, prophylactic colectomy remains controversial in the management of LS patients. Recent studies have reported that patients with CRC from LS families survived longer than did sporadic CRC patients with same-stage tumors [38, 39]. The reasons for the favorable survival rate with CRC in this syndrome remain unclear. One explanation is that immunological host defense mechanisms may be more active in tumors of the MSI phenotype [40]. Also, it has been suggested that the relatively high mutational load that occurs in tumors with defective DNA repair systems is detrimental to their survival [41].

It has been suggested that nonsteroidal anti-inflammatory drugs (NSAID) (e.g., aspirin) reduce the risk for CRC [42, 43]. Recently, Burn et al. [44] reported their results of the long-term effect of aspirin on the incidence of CRC in mutation carriers of LS. Their randomized study comprised 861 individuals with a mean follow-up time of 56 months. The authors showed that 600 mg aspirin daily clearly reduced the incidence of cancer in carriers of LS after 56-month followup. The mechanisms by which aspirin prevents the development of cancer are unknown, though some have suggested that aspirin may be proapoptotic in the early stages of CRC development [44].

## 6. Endometrial Cancer

Endometrial cancer occurs at younger ages in LS patients than in sporadic cases. The mean age at diagnosis is 49 years compared to 60 years in the general population [45]. Studies have reported that families with *MSH6* mutations are at higher risk for developing endometrial cancer (64–71%) than *MSH2* or *MLH1* families (40–50%) [24, 46]. As in the case of sporadic endometrial cancer, the majority of endometrial cancers in this syndrome are of the endometrial type [47]. Studies have shown that there is no difference in outcomes in endometrial cancer associated with LS compared to those in sporadic cancer [48].

Regular surveillance for endometrial cancer has been recommended for women with LS for the prevention or early diagnosis of cancer [34]. Some studies have investigated the efficacy of gynecological surveillance in LS. In a study by Dove-Edwin et al. [49], a study group consisted of 269 female LS family members in whom transvaginal ultrasound was performed every one to two years. No asymptomatic endometrial cancers were found, but two interval cancers were diagnosed based on symptoms. Rijcken et al. [50] presented the results of a gynecological screening program in 41 mutation carriers. They found three patients with premalignant lesions but no cases of asymptomatic endometrial cancer. A study of 175 mutation carriers from Finland reported the results of surveillance using transvaginal ultrasound and aspiration biopsy [12]. Endometrial cancer occurred in 14 cases, 11 of which were diagnosed using surveillance. Of the 11 screen-detected cancers, 6 were identified using only aspiration biopsy and not using ultrasound. The results also showed that stage distribution and survival were more favorable in the surveilled patients than in the unsurveilled patients. According to a study by Nieminen et al. [51], it seems that endometrial carcinoma will develop gradually via complex hyperplasia, and molecular genetics alternations can be observed years before cancer. Based on the previous studies [12, 49, 50], current surveillance guidelines for women with LS include annual transvaginal ultrasound and endometrial sampling starting at age 20 to 35 years. Prophylactic hysterectomy and bilateral salpingo-oophorectomy are recommended for female mutation carriers who are beyond childbearing age, especially if surgery for colorectal cancer is needed [52].

## 7. Gastric Cancer

Gastric cancer is the second most common extracolonic malignancy in LS with a cumulative risk of 2% to 13% [5, 20, 53]. As in the case of colorectal and endometrial cancers, gastric cancer in this syndrome occurs at an earlier age than in the general population [54]. The occurrence of this carcinoma seems to be not different between different mutations.

Studies have shown that over 90% of gastric cancers in LS are of the intestinal type and that tumors have a high degree of MSI [54]. Generally, the diffuse type of cancer predominates in young patients and is the most common type in the familial form of gastric cancer [55]. The development of the intestinal type of gastric cancer is closely related to *Helicobacter pylori*-associated chronic gastritis. Studies have therefore suggested that *H. pylori* infection and atrophic gastritis could serve as markers of increased risk for gastric cancer in individuals with LS. A Finnish study, however, reported that only 20% of gastric cancers in LS were *H. Pylori* positive [54].

Renkonen-Sinisalo et al. [56] evaluated the value of gastroscopic surveillance in a series of 73 mutation carriers. In this study, no cases of early cancer or premalignant lesions were detected during surveillance. A collaborative group of European experts in hereditary gastrointestinal cancer recommends screening for gastric cancer in LS families with a clustering of stomach cancer and in countries with a high prevalence of it [34]. It has been suggested that serological biomarkers such as pepsinogen I and II as well as *H. pylori* antibodies, could be a nonendoscopic screening method in evaluating individual risk for gastric cancer in LS.

## 8. Uroepithelial and Kidney Cancers

Several epidemiological studies have shown that urothelial (i.e., ureter and bladder) as well as kidney cancers are an integral part of the LS tumor spectrum with a lifetime risk of 1% to 12% [5, 6, 53]. Van der Post et al. [57] reported a clearly increased risk for bladder cancer, especially in *MSH2* mutation carriers. Correspondingly, Watson et al. [6] found that *MSH2* mutation carriers were at a sevenfold higher lifetime risk for urological tumors than were *MLH1* mutation carriers. Ureter and bladder cancers occur approximately 10–15 years earlier than those in the general population [6, 57]. The histology of these urothelial tumors associated with LS is similar to that of sporadic cases [57]. Studies have reported high frequencies of MSI for urothelial tumors in LS that clearly exceed frequencies for corresponding sporadic tumors [19].

A higher risk for kidney adenocarcinomas has been observed in a large series of LS families than in the general population [5, 6]. MSI is seldom observed in kidney cancers, however, and the median age of this cancer seems to be not lower than that in sporadic cases [19]. Therefore, researchers have suggested that kidney cancers may not be part of the tumor spectrum of LS.

Urine cytology has been shown to be insensitive in screening for bladder and ureter cancers in LS mutation carriers [58]. On the other hand, European experts in hereditary gastrointestinal cancer recommend screening for urological tumors using abdominal ultrasound, urinalysis, and urine cytology [34]. Screening should start at age 30 to 35 years with one- to two-year intervals [34]. This strategy is especially intended for families with clustering urological tumors [34].

## 9. Other Tumors

### 9.1. Ovarian Cancer

Ovarian cancer has been shown to occur in excess in LS. Two Finnish studies [5, 45] have shown a lifetime risk for ovarian cancer in LS ranging between 9% and 12% compared to 1.3% in the general population. Recently, Watson et al. [6] reported a lifetime risk of 7% in a large series from four LS research centers. They also found that *MSH2* family members had nearly twice the incidence rate observed in *MLH1* family members, and the highest risk period for ovarian cancer was from age 40 to 55 years. Ovarian cancer in LS seems to have a better prognosis than that in the general population or in BRCA1/2 mutation carriers [59]. Information currently available is too limited to assess whether screening for ovarian cancer in LS mutation carriers has any advantages.

### 9.2. Carcinomas of the Biliary Tract and Pancreas

These carcinomas seem to be associated with LS [60]. Estimates indicate that a lifetime risk of these cancers is approximately 2% in mutation carriers of LS [5, 45]. Surveillance for pancreatic or biliary tract cancer is not regularly recommended for family members with LS.

The lifetime risk for *small bowel cancer* associated with LS has been estimated to range between 1% and 4% [20, 61]. Studies have also reported that LS-associated small bowel cancer often manifests at a young age and may be the first disease manifestation [61, 62]. Tumors occur predominantly in the duodenum or the jejunum [61, 62]. Screening for small bowel cancer is not in the guidelines for clinical management of family members with LS.

### 9.3. Brain Tumors

Brain tumors occur in excess in patients with LS [63]. The estimated cumulative incidence of brain tumor ranges from 2% to 4% in family members with LS [5, 6]. Vasen et al. [53] reported that the risk for brain tumor is higher in *MSH2* than in *MLH1* mutation carriers. It has also been reported that brain tumors in LS are microsatellite stable [19]. No studies have investigated surveillance for brain tumors in LS.

## 10. Conclusions

Knowledge of the tumor spectrum in Lynch syndrome is important in planning strategies for the management of patients with this syndrome. Screening proved beneficial only for CRC and endometrial cancer although screening for other tumors is also recommended. Family history is an important tool for identifying LS. Clinical criteria serve to select suspected cases for molecular studies, such as MSI analysis of the tumors or immunohistochemical analysis of the MMR proteins. It is now possible to undertake predictive genetic testing in family members once a mutation has been detected in a family. However, it is important to organize genetic counselling individually before genetic testing. Genetic testing allows clinical screening to target mutation carriers while excluding mutation-negative individuals from further examination.
